# Beat-to-beat estimation of the continuous left and right cardiac elastance from metrics commonly available in clinical settings

**DOI:** 10.1186/1475-925X-11-73

**Published:** 2012-09-21

**Authors:** David Stevenson, James Revie, J Geoffrey Chase, Christopher E Hann, Geoffrey M Shaw, Bernard Lambermont, Alexandre Ghuysen, Philippe Kolh, Thomas Desaive

**Affiliations:** 1 Department of Mechanical Engineering, Centre for Bio Engineering at the University of Canterbury, Christchurch, New Zealand; 2 Department of Intensive Care, Christchurch Hospital, Christchurch, New Zealand; 3 GIGA-Cardiovascular Sciences, University of Liege, Liege, Belgium

## Abstract

**Introduction:**

Functional time-varying cardiac elastances (FTVE) contain a rich amount of information about the specific cardiac state of a patient. However, a FTVE waveform is very invasive to directly measure, and is thus currently not used in clinical practice. This paper presents a method for the estimation of a patient specific FTVE, using only metrics that are currently available in a clinical setting.

**Method:**

Correlations are defined between invasively measured FTVE waveforms and the aortic and pulmonary artery pressures from 2 cohorts of porcine subjects, 1 induced with pulmonary embolism, the other with septic shock. These correlations are then used to estimate the FTVE waveform based on the individual aortic and pulmonary artery pressure waveforms, using the “other” dysfunction’s correlations as a cross validation.

**Results:**

The cross validation resulted in 1.26% and 2.51% median errors for the left and right FTVE respectively on pulmonary embolism, while the septic shock cohort had 2.54% and 2.90% median errors.

**Conclusions:**

The presented method accurately and reliably estimated a patient specific FTVE, with no added risk to the patient. The cross validation shows that the method is not dependent on dysfunction and thus has the potential for generalisation beyond pulmonary embolism and septic shock.

## Introduction

Cardiac disturbances are difficult to diagnose in critical care, which can lead to poor management [1,2]. Inadequate diagnosis can be common, and plays a significant role in increased length of stay, cost and mortality [3-5]. Currently, measurements are obtained via catheters placed around the heart. However, this limited set of data can severely restrict clinical diagnostic capability, and, as a result, these catheters are not necessarily associated with improved outcomes [6-8].

Overall, much of the data currently available to clinicians in an intensive care unit (ICU) that could have significant clinical value is under utilised. For example, acute cardiovascular dysfunction, like pulmonary embolism and septic shock, severely alter cardiovascular system hemodynamics around the heart. These changes can be seen directly by catheter measurements as changes in pressure and flow that reflect dynamic changes in the balance of pre-load and afterload, resulting in an altered cardiac energetic state [9,10]. Detailed cardiac energetics are too invasive to measure in an ICU setting. However, if the relevant energetics could be captured using data from a nearby catheter, the clinical potential of such measurements could be realised. To date, no such method achieves this aim.

All models are approximations to capture observed physics, and thus all models offer advantages in understanding these observations as well as limitations of any necessary assumptions made. This paper presents a model for estimating the functional time-varying elastance from observed ventricular pressure-volume behaviour, to be used in the modelling of broader cardiovascular system [11-15]. Thus, this is the elastance required to deliver the pressure-volume measurements observed, which implicitly contain the pre-load and afterload information. This is in contrast to the “true” elastance which is a physical property of the heart muscle alone and can be tested only ex-vivo. To make this distinction clear, the elastance presented in this paper is referred to as the “functional elastance”.

Functional time-varying cardiac elastance (FTVE) is defined as [16]:

(1) $$e(t)=\frac{P_{v}(t)}{V_{v}(t)-V_{d}}$$

where, *V*_*d *_is assumed to be equal to *V*_0 _for simplicity, *V*_0 _is the intercept of the end-systolic pressure-volume relation with the volume axis [17], *P*_*v *_(*t*) is the ventricle pressure and *V*_*v *_(*t*) is the ventricle volume. The waveform *e*(*t*) is typically normalised to a value of 1.0 [18]. This model of load-dependent functional time-varying elastance should not be confused with the measurement of end-systolic elastance, which is regarded as load insensitive within physiological range. However, the load-independence of end-systolic elastance is an approximation made to that model, as the true end-systolic elastance is dependent on loading condition, if only weakly within physiological range [19]. Load conditions are diagnostic and thus the model for FTVE presented monitors these changes as the time varying ability of the heart to pump blood. It thus provides a measure of heart function and energetics [18,20,21].

There have been several attempts to estimate FTVE [20,22-25]. Most studies present a method using the FTVE to estimate a specific parameter, most commonly end-systolic elastance [20,24,25] and ejection fraction [23]. However, their validation is based on these metrics, not on the resulting FTVE waveform, which contains unique clinical information. This study is focused not on the absolute values of the elastance (such as end-systolic elastance). Rather, the focus is on the shape, and change of shape of the time-varying waveform within a specific patient as dysfunction occurs and the patient’s state changes. This intra-patient variability is reflected in the (relative) shape.

It is unclear how much specific clinical information can be obtained from the FTVE waveform, other than the highly sought-after end-systolic elastance [26], and a measure of cardiac work (the area under the FTVE waveform is analogous to work done by the ventricle). The absolute value of the work done is lost with the normalisation of the waveform, but the relative changes to work over time remain. This holds true for a constant inotropic state, as the end-systolic elastance will remain the same. For a varying end-systolic elastance, the relative change in cardiac power will still be visible to the FTVE through the altered shape from an increased heart rate.

Beyond cardiac work a number of FTVE features have been highly correlated to clinically relevant parameters in a previously developed circulatory model [27]. Equally, it contains similar information to that of the pressure-volume (P-V) loops, which are known to contain information on cardiac function [28] including cardiac work [29,30], contractility [18,31], O_2 _consumption [30,32], and all the states of filling, contraction ejection and relaxation [1]. Overall, cardiac elastances reflect cardiac state, cardiac output or blood volume, and net pre-load and afterload, all of which change with different cardiac dysfunction. Hence, the ability to directly measure FTVE, which does not currently exist without significantly invasive added testing, should yield clinically useful insight and diagnostics.

This research is unique in that the end goal is to produce the FTVE function in its own right, validating the FTVE waveform on its own accuracy compared to the invasively and directly measured waveform. This is done through the use of only commonly available metrics in an ICU setting, and thus, by non-invasive we mean, no more invasive than traditional care.

## Methods

### Concept

Cardiac elastance cannot be directly measured without invasive procedures. However, the information contained within the cardiac elastance waveform can be seen in other parts of the closely intertwined cardiovascular system. Therefore, with the right knowledge and transformations, that information can be collected and translated into an estimation of a metric (cardiac elastance) that would not normally be possible to obtain.

The best accessible source of indirect information about cardiac elastance is directly down stream of the ventricles at the aorta and the pulmonary arteries. They are the first to see any results of a change in elastance during systole. Thus, the aortic and pulmonary artery pressure waveforms (*P*_*ao *_and *P*_*pa*_, respectively) are key sources of information, which contain surrounding load conditions, to reconstruct cardiac elastances. It can be assumed, and seen from the data presented in this paper, that the shape of the functional time-varying cardiac elastance is load dependent.

**Figure 1:**
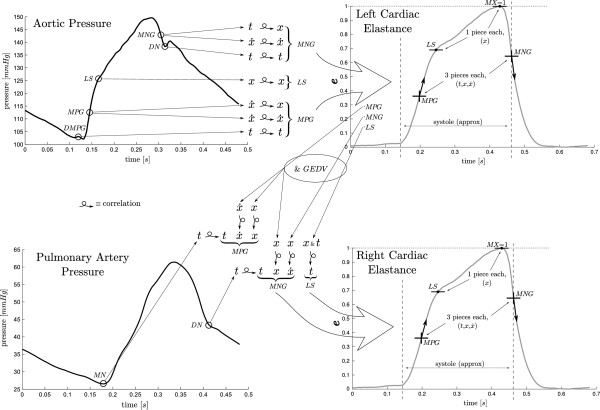
**Overview. **An overall description of the method presented in this paper, showing the left and right FTVE waveforms (the right half of the figure), with the four points required to reconstruct them, and the pressure waveforms (the left half of the figure) which are used to generate the correlations and the final approximations. The large hollow arrows show the general flow of information, while the smaller arrows show more fine grained flow. The left ventricle FTVE (top right) is estimated solely from the shown points on the aortic pressure, while the right ventricle FTVE (bottom right) is estimated from the estimation of the left ventricle FTVE, along with timing from the pulmonary artery pressure and GEDV.

For describing the methods in this paper, a naming convention from Figure 1 is defined:

(2) $$\begin{array}{lll} P_{\mathrm{ao}} & \equiv \text{aortic pressure}\; & \;\\ P_{\mathrm{pa}} & \equiv \text{pulmonary artery pressure}\; & \;\\ \text{DMPG} & \equiv \text{driver maximum positive gradient}\; & \;\\ \mathrm{MN} & \equiv \text{minimum point}\; & \;\\ \text{MPG} & \equiv \text{maximum positive gradient}\;\\ \mathrm{LS} & \equiv \text{left shoulder}\; & \;\\ \text{MNG} & \equiv \text{maximum negative gradient}\; & \;\\ \mathrm{DN} & \equiv \text{dicrotic notch}\; & \;\\ e(t) & \equiv \text{functional elastance curve}\; & \; \end{array}$$

Figure 1 shows the overall method of this paper. Known points on the aortic pressure waveform are correlated with points on the left ventricle FTVE (described in Section “Cardiac elastance correlations”), giving equations that enable estimations of these left ventricle points from the aortic pressure alone. A function is then used to draw a smooth line through these estimated points (described in Section “Waveform construction”) giving a continuous approximation of the left ventricle FTVE. The right ventricle is estimated in similar way. Known points from the estimated left ventricle FTVE, time values taken from the pulmonary artery pressure, and the global end-diastolic volume (GEDV) are correlated with points from the right ventricle FTVE, again creating equations used to estimated a continuous right ventricle FTVE.

**Figure 2:**
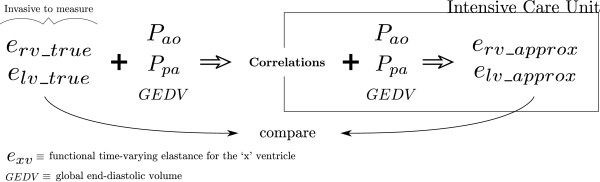
**Research vs clinical use. **This research proposes a method which contains two sides. One research side which involves the preparation of the correlations from invasively measured metrics (shown on the left of the figure), and a clinical method for use in an ICU, where, the defined correlations are used with metrics already available in an ICU to estimate the cardiac elastance (shown in the box).

A total of 8 pieces of information (within the four labelled points on cardiac elastance waveforms) are needed to draw a smooth curve for the estimated cardiac elastance. These points (*MNG*, *LS*, *MPG* and *MX*) are shown on the FTVE waveforms in Figure 1 and to the left of the respective FTVE (with the exception of *MX* as this is always 1), along with a break down of the origins of their values. The points on the pressure waveform required in this method (from (2)) are shown on the aortic and pulmonary artery pressure waveforms. The large hollow arrows show the overall flow of information within the method, while the smaller arrows show the more fine grained flow of information. A simpler model is shown in Figure 2, which visually shows the difference between the academic side described in this paper and the clinical usage of this research.

It should be noted that the two catheter locations (aorta and pulmonary artery) can only “see” the ventricles during systole, inferring that information about the cardiac elastance can only be known during systole. However, this limitation is not clinically significant as cardiac elastance is primarily defined by what happens during systole.

### Animal data

The method presented was developed on a cohort of 9 porcine subjects divided into two groups. The first group was induced with pulmonary embolism, (5 subjects) [33,34]. The second group (4 subjects) were induced with septic shock [35]. Both trials were under the control of the ethics committee of the medical faculty of Liege, Belgium.

Measurements were taken every 30 minutes. In total the first group had 51 sets of measurements across the 5 pigs, while the septic shock cohort had 34 over 4 pigs. For each pig, the following relevant measurements were taken: *P*_*ao*_, *P*_*pa*_, left and right ventricle volume (*V*_*lv*_, *V*_*rv*_), left and right ventricle pressure (*P*_*lv*_, *P*_*rv*_). Aortic and pulmonary artery pressure were measured using catheters, while right and left ventricle pressures and volumes were directly measured using 7F, 12 electrodes conductance micro manometer tipped catheters.

Left and right cardiac elastances were calculated directly with the respective pressure and volume waveforms via (1), while setting the dead space volume to zero. GEDV was calculated as the sum of the individual maximum volumes of the left and right ventricles.

The data contains varying degrees of cardiac dysfunction from healthy to the fully developed disease state. It thus provides a good test of the presented methods. The use of two dysfunction states is designed to illustrate the method’s potential robustness.

### Waveform construction

**Figure 3:**
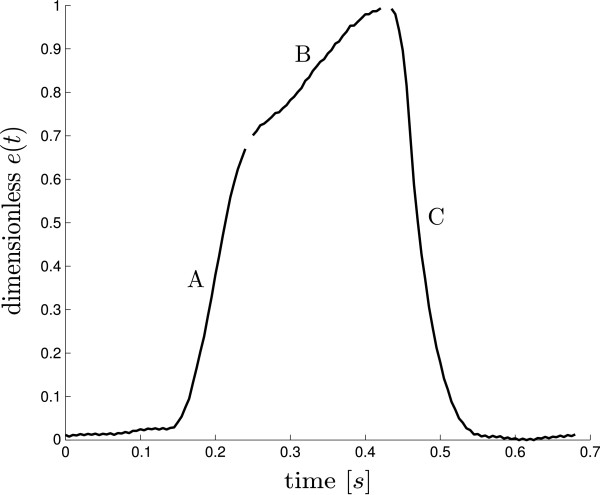
**Sectioned cardiac elastance waveform. **An example of a typical cardiac elastance for the left side of the heart broken into its main sections, an exponential rise (**A**) and decay (**C**) and a near straight section in between (**B**).

The general shape of the cardiac elastance has three main sections: an exponential rise (A), a shoulder section (B) and an exponential decay (C), as shown in Figure 3. An invasive vena cava occlusion maneuver is assumed to be not available and thus *V*_*d *_= 0 in (1). The cardiac elastance is normalized to 1.0 [18].

The shape of the cardiac elastance waveform is formed from 3 known locations on the waveform; time, slope and height of the maximum rising gradient ($t_{1},{\dot{x}}_{1},x_{1}$) and maximum falling gradient ($t_{3},{\dot{x}}_{3},x_{3}$), (analogous to *MPG *and *MNG *respectively in Figure 1), and the height of the waveform^*′*^s left shoulder, *x*_2_, (analogous to *LS*, in Figure 1). These are 7 of the 8 values needed to reproduce the waveforms. The eighth is the height of the right shoulder (*MX*, in Figure 1) which is always unity. The 7 variable values are found with correlations from points on the *P*_*ao *_and *P*_*pa *_waveforms, as described in Section “Cardiac elastance correlations”.

The FTVE waveform is assumed to closely fit two exponential functions (one rise and one decay) joined by a straight line and *e*(*t*) is thus defined:

(3) $$e(t)=\left\{\begin{array}{c} F_{\alpha }(t)\;\;\;\;\;0<t<c_{\alpha }\\ \frac{\left(1-x_{2})(t-c_{\alpha }\right)}{c_{\beta }-c_{\alpha }}+x_{2}\;\;c_{\alpha }<t<c_{\beta }\\ F_{\beta }(t)\;\;\;\;\;c_{\beta }<t<\mathrm{period} \end{array}\right)$$

where:

(4) $$F_{i}=a_{i}\cdot e^{-b_{i}{\left(t-c_{i}\right)}^{2}}$$

and the coefficients of (4), also seen in (3), are fitted for a specific waveform, and are defined:

(5) $$\begin{array}{ll} a_{\alpha } & =x_{2}\\ b_{\alpha } & =-\frac{\text{log}(x_{1}/x_{2})}{\text{exp}(\text{log}(-\text{log}(x_{1}/x_{2})\cdot 2\cdot (x_{1}/{\dot{x}}_{1}))\cdot 2)}\\ c_{\alpha } & =-\frac{\text{log}(x_{1}/x_{2})\cdot 2\cdot x_{1}-{\dot{x}}_{1}\cdot t_{1}}{{\dot{x}}_{1}} \end{array}$$

where *a*_*β*_, *b*_*β *_and *c*_*β *_are similarly defined by replacing subscript 1 with 3 and setting *x*_2 _= 1.

### Cardiac elastance correlations

The right and left ventricle elastances are constructed using Equations (3) to (5). However, the 8 values that identify the separate elastances are found in different ways.

Table 1 shows three different types of correlations, the first of which (*α*) is the standard correlation used in most statistical analysis. The second two types, *β* and *γ*, are extensions to two and three variables, respectively.

**Table 1:** Reconstruction formulae for the three different types of correlations used to estimate the cardiac elastance

**Key**	**Re-construction formula**
*α*	*y *= *mx *+ *c*
*β*	*y *= *m*_1 _*x*_1 _+ *m*_2 _*x*_2 _+ *c*
*γ*	*y *= *m*_1 _*x*_1 _+ *m*_2 _*x*_2_+ *m*_3 _*x*_3 _+ *c*

The left cardiac elastance values are relatively straightforward to find, and all use the standard correlations, *α*, from Table 1. The right cardiac elastance is slightly harder to estimate, partly because there is no direct down stream access to the right ventricle similar to that of the left ventricle. This is because only timing information is taken from *P*_*pa*_, which can be replaced with electrocardiography (ECG) and central venous pressure (CVP), as the *P*_*pa *_waveform is not commonly measured in an ICU. This self imposed limitation makes the method more clinically applicable.

This limitation necessitates other means to estimate the required values on the right side, which is mostly done through the use of multi-variable correlations involving GEDV, and the estimated left cardiac elastance. GEDV is closely connected to the average volume in the right ventricle, and thus serves to add additional scaling. The specific correlations found of the left and right elastances are shown in the Results.

### Error calculations

**Figure 4:**
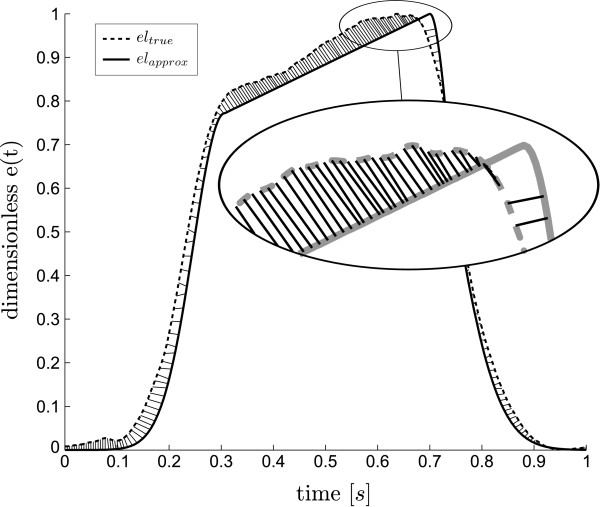
**Cardiac elastance error metric. **An example of the calculation of the error metric. At each point on the true waveform, the nearest point on the estimated waveform is located, and the distance between these two points is calculated (draw as solid lines). This is an approximation of the normal distance between the two waveforms. From this series of errors values along a waveform, median and 90^th ^percentile errors are calculated.

The error of the estimated cardiac elastance waveform is defined using a distance metric between the true *e*_*true *_and estimated *e*_*est *_waveforms, after both waveforms have been normalised to the heart rate (producing a waveform confined in a 1×1 box), which allows the error metric to be consistent in all directions. This error metric is chosen instead of a more standard root mean square error (RMSE) to more accurately reflect the error between the waveforms. Due to the shape of these waveforms, a RMSE would over emphasise the error due to any slight timing mismatch in the steep sections of the waveform. The error at each point on the approximated waveform is defined:

(6) $$\delta_{i}=\underset{t\in [0,1]}{\text{min}}\left\{\sqrt{{\left(t_{i}-t\right)}^{2}+{\left(e_{\text{est}}(t_{i})-e_{\text{true}}(t)\right)}^{2}}\right\},\;i=1,\ldots N$$

where *N *is the number of discrete points on the approximated waveform. The errors of the estimated waveforms are represented by a median and a 90^th^ percentile of the values of *δ*_*i *_in (6), for both single waveforms and whole cohort errors. Where multiple waveform are involved in a single error metric (such as those of the whole cohort), the *δ*_*i *_values are concatenated into a single vector before the median and percentile errors are calculated. The values of *δ*_*i *_in (6), geometrically correspond to the distance from the point (*t*_*i*_, *e*_*est *_(*t*_*i*_)) to the closest point in the curve {(*t*, *e*_*true *_(*t*)), *t*∈[0,1]}, as illustrated in Figure 4. For practicality of computation, the real waveform is discretised, such that the number of points is greater than *N*. Due to this discretisation the error is over estimated, as the line between the two points is not the exact normal line of the point on the estimated waveform. The approximation has been chosen over the exact calculation due to the fact that for a given point on the estimated waveform there is no guarantee that a point exists on the real waveform that is exactly normal to it.

### Analyses

The cardiac elastances were estimated for every measurement taken at 30 minute intervals during the onset of the porcine cohort’s cardiac dysfunction, in two different ways. First, using the correlations created using the entire cohort of 9 pigs (both dysfunctions). Second, using the correlations determined only from the five (or four) pigs from the “other" dysfunction. These estimations were then evaluated against the true cardiac elastance to determine the error, as in (6).

## Results

### Correlations

**Figure 5:**
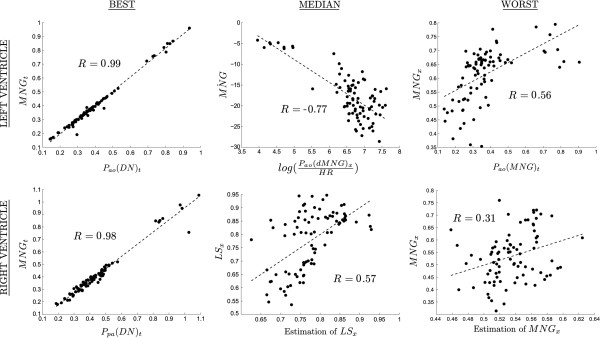
**Example correlations. **Three correlations are shown for each of the left (top row), and the right cardiac elastance (bottom row). These three represent the best (left), median (middle) and worst (right) correlations by *R* value. The median and worst case for the right cardiac elastance are multi-variable correlations, and hence only a visualisation, so they cannot be used to read off data in the way a single variable correlations graph can.

The correlations on both left and right sides, as defined in Figure 1, were good. The *R* values can be found in Tables 2 and 3 for the left and right, respectively. The best, median and worst correlations by *R *value are visualised in Figure 5. The left side correlations are very strong, especially those estimating time. The right side correlations are weaker than the left side due to the approximations made by not using *P*_*pa *_pressure and flow values, which increases clinical applicability. However, they are still strong enough to produce very accurate overall estimations of the continuous right cardiac elastance.

**Table 2:** **Left ventricle cardiac elastance correlations, all points use correlation *α *from Table **1

**Estimate**	**Correlated to**	**R value**	**Coefficients**
*MPG*_*x *_(*x*_1_)	$\frac{P_{\mathrm{ao}}{\left(\text{MPG}\right)}_{\dot{x}}}{\mathrm{HR}}$	0.71	*m *= 2.521×10^−5^
			*c *= 3.732×10^−1^
*MPG*_*t *_(*t*_1_)	*P*_*ao *_(*DMPG*)_*t*_	0.99	*m *= 1.007
			*c *= 1.615×10^−3^
$M\dot{P}G({\dot{x}}_{1})$	$\frac{P_{\mathrm{ao}}(\text{MPG})\dot{x}}{\mathrm{HR}}$	0.94	*m *= 4.126×10^−3^
			*c *= 8.665
*MNG*_*x *_(*x*_3_)	*P*_*ao *_(*MNG*)_*t*_	0.56	*m *= 3.370×10^−1^
			*c *= 4.886×10^−1^
*MNG*_*t *_(*t*_3_)	*P*_*ao *_(*DN*)_*t*_	0.99	*m *= 1.028
			*c *= −9.148×10^−3^
$M\dot{N}G({\dot{x}}_{3})$	$\text{log}\left(\frac{\text{Pao}{\left(\text{MNG}\right)}_{\dot{x}}}{\mathrm{HR}}\right)$	−0.77	*m *= −5.510
			*c *= 1.871×10^ + 1^
*LS*_*x *_(*x*_2_)	$\frac{P_{\mathrm{ao}}{\left(\mathrm{LS}\right)}_{x}}{\mathrm{HR}\cdot MX_{x}}$	0.74	*m *= 9.627×10^−2^
			*c *= 5.867×10^−1^

**Table 3:** Right cardiac elastance correlations

**Estimate**	**Correlated to**	**R**	**Type**	**Coefficients**
*MPGs*_*x *_(*x*_1_)	*GEDV*, *e*_*lv *_(*MPG*)_*x*_	0.42	*β*	*m*_1 _= −1.137×10^−3^
				*m*_2 _= 3.521×10^−1^
				*c *= 4.524×10^−1^
*MPG*_*t *_(*t*_1_)	*P*_*pa *_(*MN*)_*t*_	0.97	*α*	*m *= 1.023
				*c *= −4.102×10^−3^
$M\dot{P}G({\dot{x}}_{1})$	$\text{GEDV},e_{\mathrm{lv}}{\left(\text{MPG}\right)}_{\dot{x}}$	0.80	*β*	*m*_1 _= −1.286×10^−2^
				*m*_2 _= 6.446×10^−1^
				*c *= 6.910
*MNG*_*x *_(*x*_3_)	*GEDV*, *e*_*lv *_(*MNG*)_*x*_	0.31	*β*	*m*_1 _= 1.233×10^−3^
				*m*_2 _= 1.824×10^−1^
				*c *= 2.348×10^−1^
*MNG*_*t *_(*t*_3_)	*P*_*pa *_(*DN*)_*t*_	0.98	*α*	*m *= 9.751×10^−1^
				*c *= −2.081×10^−2^
$M\dot{N}G({\dot{x}}_{3})$	*GEDV*, *e*_*lv *_(*MPG*)_*x*_	0.37	*β*	*m*_1 _= −6.096×10^−2^
				*m*_2 _= 1.446
				*c *= −4.331
*LS*_*x *_(*x*_2_)	*GEDV*, *e*_*lv *_(*LS*)_*x *_,*e*_*lv *_(*LS*)_*t*_	0.57	*γ*	*m*_1 _= −2.054×10^−3^
				*m*_2 _= 3.209×10^−1^
				*m*_3 _= −3.538×10^−1^
				*c *= 9.045×10^−1^

### Cardiac elastances

**Figure 6:**
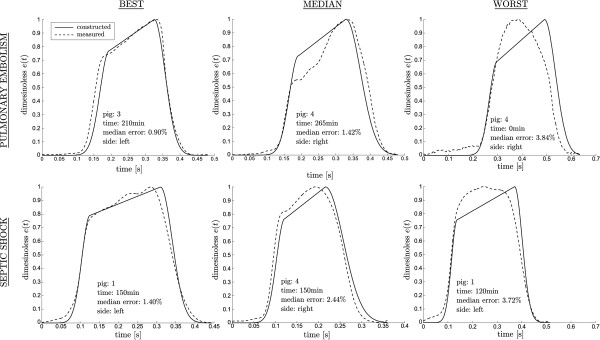
**Example results. **Results of the estimation of the cardiac elastance alongside the corresponding measure elastance, for both pulmonary embolism (top row) and septic shock (bottom row). For both conditions the 10th, 50th and 90th percentile case by median error are shown in positions left, middle and right respectively.

The cross testing (described in section “Analyses”) ensures robustness and independent validation. The results comparing directly measured *e*(*t*) to estimated waveforms are listed in Tables 4 and 5 for both the correlations using all data, and for the independent cross validation using only correlations from the ‘other’ dysfunction. The estimated waveforms are illustrated in Figure 6, with the 10^th^, 50^th ^and 90^th ^percentile (by percentage error) estimated results. Table 4 shows the errors for the same estimated waveforms for pulmonary embolism and septic shock, in terms of the percent error between clinically measured and the estimated driver function.

**Table 4:** Reconstruction errors for pulmonary embolism and septic shock

	**Pulmonary embolism**		**Septic shock**	
	**Left**	**Right**	**Left**	**Right**
median	1.10%	2.10%	2.03%	2.87%
90th percentile	3.35%	6.53%	5.62%	8.65%

**Table 5:** Cross validation reconstruction errors for pulmonary embolism and septic shock

	**Pulmonary embolism**		**Septic shock**	
	**(based on septic shock)**		**(based on PE)**	
	**Left**	**Right**	**Left**	**Right**
median	1.26%	2.51%	2.54%	2.90%
90th percentile	4.87%	7.07%	8.25%	9.30%

Table 5 shows the errors using the correlations derived from one dysfunction to approximate the driver function of the ‘other’ dysfunction. This last assessment cancels out any inherent dependence of the method, outlined in this paper, on the dysfunction itself and is a rigorous robustness test. The results show the method is independent of dysfunction, in this case, which is an extremely important result. For a method to reliably give insight into a specific patients cardiac dysfunction, it must not rely on the patient having a specific cardiac dysfunction. It is also another validation of the accuracy and potential of this method, which gives further confidence that the method will generalise over a wider set of dysfunctions.

**Figure 7:**
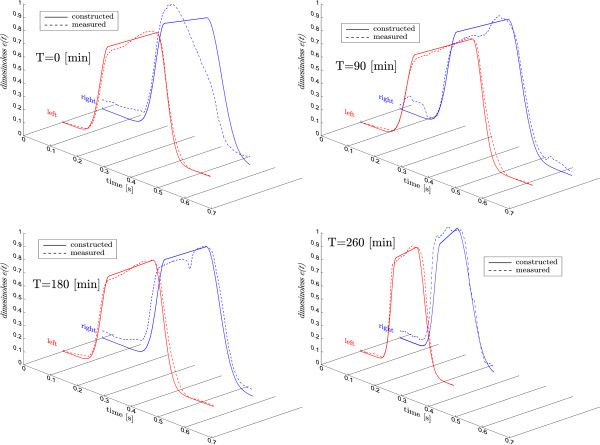
**Left and right cardiac elastance results. **Four reconstructions of the left and right driver function, from the same pig as the pulmonary embolism progresses from healthy at *t *= 0 to the end of trial at *t *= 260.

Figure 7 shows four reconstructed driver functions versus the directly measured driver functions, for pig four at four different times during the onset of pulmonary embolism. In particular, the dramatic decrease in period at *t *= 260 min, which is captured very well. Overall, the trends and basic shape are captured very well for the left and reasonably well for the right.

## Discussion

A method for identifying time varying elastance of the left and right ventricles, was developed that doesn’t require an occlusion manoeuvre to find *V *_*d*_, and avoids invasive measurements of left and right ventricle volumes and pressure. These time varying elastance functions can be used as the input driver function into a lumped parameter cardiovascular model. The method tracks the main, clinically important time features on the driver functions, such as the start and end of ejection. This method was validated on two sets of porcine data, which included multiple measurements of five pigs that were induced with pulmonary embolism.

The approach taken to chose the mapping from the known metrics to the cardiac elastance, was based both on physiology and accuracy of the resulting cardiac elastance waveform. Thus some mappings may not be the most obvious choice based on purely physiological reasoning. Some of these mapping may appear to be counter-intuitive. For example, although *MPG *and *MNG *appear to have symmetry of meaning in the aortic pressure waveform, this does not transfer into symmetrical meaning for cardiac energetics, and hence do not have symmetry in the chosen mappings.

The correlations that were used in the reconstructions presented in this paper are generally strong. The time based correlations are especially strong (see the 2 left most plots in Figure 5), and lead to very good estimations of the waveform timing. There are also some rather poor correlations, the worst of which predicts the value of the right ventricle FTVE at the point *MNG*. This correlation has *R *= 0.31, and a correlation plot that barely shows any correlation at all. However, the final estimations of the waveform are not very sensitive to this value, and hence can produce reasonably accurate waveform estimations from values of *MNG*_*x *_that error prone. Note that the main results for this paper are the errors of the reconstructed waveforms, rather than the presence of correlations.

There is also a marked difference in the correlation’s *R *values between the left and right sides. This is mostly due to not have direct access to the right side of the heart (since all but the timing of the *P*_*pa *_waveform have been neglected), and instead are inferring information about the right side from the left side. Although this results in significantly lower *R *valued correlations (compared to the left), the waveform reconstructions are still good, with the errors averaging only 5% higher than the left side, for the cross dysfunction validation.

As would be expected from the correlations in Table 2, the timings of the reconstructed waveforms were very good, capturing the ascending and descending exponential sections very closely across a large change in cardiac condition, including an approximate doubling in heart rate. The biggest difference in the real and reconstructed waveforms usually appeared in the flat section (B of Figure 3). This error could be reduced by using a higher order polynomial or model. However, simulation has shown that this error does not have a significant effect on outputs of the existing cardiovascular model [12]. In addition the philosophy of this research is to only add complexity as required to capture and predict clinical data, and to keep the model as simple as possible. The straight line approach would also have the benefit of simplifying any semi-analytical solution approach to speeding up the simulations which is planned in future work [36].

The specific waveform for FTVE was chosen as a balance between accuracy and complexity. A model (waveform shape) was needed that provided enough information to capture the necessary cardiac dynamics, while remaining as simple as possible. The simplest waveform shape is that of three straight lines. To fit this model, four points would be required (or equivalently, two points with associated gradients and two heights (points with free ‘x’ coordinate) - the currently used data, see Figure 1). However, for the same amount of information, and no additional computational complexity, a model of two exponential curves and one straight line (the chosen model) can be fitted. Furthermore, this model is continuously differentiable, which can be computationally advantageous in numerical simulation. This more complex shape, provides more accurate results, without adding complexity to the process of fitting. Beyond this model structure, more complex models would require additional information to be found from the pressure waveforms.

For simplicity of the model, *V *_*d *_was set to zero, which is a limitation of the current approach. However, even with this discarded parameter, the model is still able to capture the necessary dynamics, and provide useful additional information to an ICU clinician base on the trends observed in FTVE shape relative to this assumption. Having an accurate *V *_*d *_value in addition would add useful information, but is not identifiable from the data available without added invasive sensors.

Another limitation of the paper is the small number (*N *= 9) of porcine subjects (although there were a large number (*N *= 85) of data sets used) and only two dysfunctions. However, this paper serves as a proof of concept as its main goal. In particular, the results are intended to justify the methods and thus a larger, complete prospective validation.

Although this study used the pulmonary artery pressure, the points found in the pulmonary artery pressure waveform could be estimated using the central venous pressure and ECG. Thus, there is the potential to construct the driver functions without any knowledge of the right side of the heart. This option would avoid using pulmonary artery pressure, which is less commonly available than aortic pressure in an ICU. This study did not have access to CVP and ECG data for the porcine cohort so used pulmonary artery pressure instead. Future work will investigate these clinically relevant extensions to minimise invasiveness, including the application of the methods onto ICU patients.

## Conclusions

This paper describes a method that has huge clinical potential. Currently, disease states like pulmonary embolism and septic shock are difficult to diagnose accurately and reliably due to the lack of useful information accessible to the clinician. Knowing the continuous cardiac elastance can gather this otherwise under utilised information into a more clinically applicable data set. This is achieved through a method that uses only currently available metrics in the ICU, and thus is no more invasive (no additional risk) than standard clinical practice. Preliminary results for pulmonary embolism indications from the cardiac elastance show promise.

## Competing interest

The authors declare that they have no competing interests.

## Authors’ contributions

DS drafted the manuscript and developed the algorithm. JR and JC participated in the algorithm development with added input from TD. CH participated in the initial mathematical formulation. GS provided physiological understanding and clinical input at all stages. BL, AG, PK and TD provided the porcine data and further clinical input and relevance. JC and TD edited and aided the writing of the manuscript and revisions with DS. All authors read and approved the final manuscript.
